# An annotated test-retest collection of prostate multiparametric MRI

**DOI:** 10.1038/sdata.2018.281

**Published:** 2018-12-04

**Authors:** Andriy Fedorov, Michael Schwier, David Clunie, Christian Herz, Steve Pieper, Ron Kikinis, Clare Tempany, Fiona Fennessy

**Affiliations:** 1 Brigham and Women’s Hospital, Harvard Medical School, Boston, MA, USA; 2 PixelMed Publishing, Bangor, PA, USA; 3 Isomics Inc., Cambridge, MA, USA; 4 Fraunhofer MEVIS, Bremen, Germany; 5 Mathematics/Computer Science Faculty, University of Bremen, Bremen, Germany

**Keywords:** Diagnostic markers, Cancer imaging, Prostate

## Abstract

Multiparametric Magnetic Resonance Imaging (mpMRI) is widely used for characterizing prostate cancer. Standard of care use of mpMRI in clinic relies on visual interpretation of the images by an expert. mpMRI is also increasingly used as a quantitative imaging biomarker of the disease. Little is known about repeatability of such quantitative measurements, and no test-retest datasets have been available publicly to support investigation of the technical characteristics of the MRI-based quantification in the prostate. Here we present an mpMRI dataset consisting of baseline and repeat prostate MRI exams for 15 subjects, manually annotated to define regions corresponding to lesions and anatomical structures, and accompanied by region-based measurements. This dataset aims to support further investigation of the repeatability of mpMRI-derived quantitative prostate measurements, study of the robustness and reliability of the automated analysis approaches, and to support development and validation of new image analysis techniques. The manuscript can also serve as an example of the use of DICOM for standardized encoding of the image annotation and quantification results.

## Background & Summary

Multiparametric Magnetic Resonance Imaging (mpMRI) is an effective tool in detecting and characterizing prostate cancer (PCa)^1^. Interpretation of the mpMRI in the clinic is done largely using the Prostate Imaging - Reporting and Data System (PI-RADS)^2^ - a score-based system that relies on qualitative interpretation of the image with the goal of identifying clinically significant PCa. Numerous research studies have demonstrated the promise of quantitative mpMRI that aims to improve upon subjective qualitative assessment. Imaging-based measurements derived either from operator-specified regions, or relying on automatic localization of the disease have been studied for a wide range of PCa applications from automated characterization^3^, to non-invasive monitoring of progression^4^ and evaluation of treatment response^5,6^. Further characterization of this early evidence and the implementation of quantitative imaging procedures more broadly requires improved understanding of the technical robustness and reliability of mpMRI as an imaging biomarker of PCa. One such aspect of technical assessment of a prospective biomarker involves evaluation of its repeatability, i.e., characterization of the consistency of the measurement under similar conditions in absence of underlying changes in the tissue biology. One of the approaches to evaluating repeatability involves imaging of subject multiple times over a short interval of time using identical imaging equipment. This type of study enables evaluation of acquisition-related sources of variability in absence of biological changes in the tissue, and is known as test-retest.

Although mpMRI is being widely investigated as an imaging biomarker of PCa^3,4,7,8^, little is known about its repeatability. Litjens et al. investigated repeatability in a study that involved repeated imaging of subjects within a longer interval of 6-12 months (n = 10)^9^. Gibbs et al. studied the repeatability of apparent diffusion coefficient in healthy subjects (n = 8)^10^. There are also studies investigating short-term repeatability of mpMRI (repeated imaging done within minutes, with or without repositioning of the subject within the MRI scanner)^11,12^. In an attempt to bridge this important gap in knowledge, we recently conducted analysis of PCa mpMRI in a test-retest study that investigated repeatability of measuring volume and mean Apparent Diffusion Coefficient (ADC) in small volume suspected disease, and anatomical regions of interest defined manually^13^. The study involved 15 subjects, and was the first to investigate short term repeatability of 3 T mpMRI obtained with the use of endorectal coil (imaging obtained within the period of 2 weeks, involving repositioning of the patient and imaging not done on the same date).

None of the prostate mpMRI repeatability studies published to date is accompanied by the imaging datasets used to arrive at the study findings. This prevents the opportunities for reusing the dataset to better understand the findings, or to apply the data to new studies. As an example, it is logical to expect, and there is evidence in other test-retest studies, that repeatability of volume measurement is dependent on the lesion size^14^, but subject-level information on the lesion volume is not available for any of the published studies. Applications of radiomics^15^ — quantitative characterization of radiographic phenotype using a variety of automatically derived metrics, such as texture and shape descriptors — is of increasing interest as applied to PCa^16^. No studies have investigated so far the repeatability of radiomic features, or the effects of various processing parameters on their stability.

In the present data descriptor we share the original imaging dataset that was analyzed in our earlier study^13^. The imaging data we collected is accompanied by the image annotations and derived measurements used to quantify repeatability of volume and mean ADC in the earlier published manuscript. Some of the uses of the dataset we envision include further investigation of additional aspects of repeatability (e.g., as applied to radiomic features, investigation of pre-processing approaches that could improve consistency of image-based measurements), development of new algorithms (e.g., automatic image segmentation, longitudinal image registration), and quantification of technical capabilities of the automatic processing tools (e.g., consistency of lesion detection, consistency of automatic segmentation and registration, evaluation of computer-assisted tools for automated detection and characterization of PCa).

The resulting dataset (not just imaging data, but also image annotations and image-derived measurements) is shared as a collection of objects that are encoded using standardized Digital Imaging and Communications in Medicine (DICOM) (ISO 12052:2017) representation^17–19^ (https://www.dicomstandard.org/). This continues the precedent we established earlier to utilize standard representation for harmonizing data as applied to quantitative imaging biomarker development^20^.

## Methods

The Institutional Review Board of the Brigham and Women’s Hospital approved the Health Insurance Portability and Accountability Privacy Rule compliant study in which the image dataset was collected. Informed consent was obtained from all participants. Permission to share the de-identified imaging data, annotations and derived measurements in a restricted-access collection of The Cancer Imaging Archive (TCIA)^21^ was obtained from the Institutional Review Board (IRB) of the Brigham and Women’s Hospital. The data is stored in the QIN-PROSTATE-Repeatability collection of the TCIA (see Data Citation 1).

The reader is referred to the manuscript summarizing the study and the analysis of the data for the study details^13^. Here we provide an abbreviated summary of the data acquisition details presented earlier, and discuss the dataset itself in the subsequent sections.

Magnetic resonance imaging exams of the abdomen were collected for 15 men at the Brigham and Women’s Hospital (BWH). All of the subjects scanned were treatment-naive at the time of imaging. The cohort included both patients with the histologically confirmed PCa, and patients with no definite diagnosis that had a reason to be evaluated for the presence of PCa. The time interval between the baseline and repeat imaging exam was within 2 weeks. Both of the mpMRI exams were done using the standard clinical protocol implemented at BWH. Patients were scanned on 3 Tesla field strength scanners, either GE Signa HDxt or GE Discovery MR750w. In all cases, the baseline and repeat study for a given patient was done using the same scanner, with the use of a commercially available endorectal coil within an air-filled balloon (Medrad Inc, Warrendale, PA). All of the acquisition parameters are available within the DICOM MR images included in the collection.

Acquired mpMRI data was annotated by a radiologist (further referred to as “the reader”) with over 10 years of experience in abdominal MRI interpretation (F.F.). Annotation was performed using the 3D Slicer research platform^22^ (https://slicer.org) and involved planimetric labeling of the voxels within the regions of interest (ROIs) corresponding to the entire prostate gland and peripheral zone of the prostate, as well as tumor and normal areas of the peripheral zone (suspected tumor was identified in 11 of the 15 subjects). Each of the structures was annotated separately for each of the subjects using contouring tools in the 3D Slicer Editor module in both baseline and repeat studies for the T2-weighted image series, Apparent Diffusion Coefficient (ADC) maps derived from the Diffusion-Weighted acquisition that utilized b-values of 0 and 1400 mm/s^2^, and the subtracted images derived from the Dynamic Contrast-Enhanced (DCE) acquisition. The individual studies were randomized such that the reader was blinded to the identification of the subject for a given study, or whether that study was a baseline or a repeat exam. While annotating a given study, the reader reviewed all of the aforementioned sequences side-by-side, localizing the suspected tumor region by evaluating characteristics based on consensus of its appearance in individual sequences. This approach followed the PI-RADS v2 guidelines^2^, although the encoding of PI-RADS reports is not part of the present dataset. Once localized, the region was contoured in each of the individual image series based solely on its appearance in that image. Regions were annotated in the individual sequences, since it is known that separate MRI sequences interrogate different attributes of the disease, and the goal of the original data analysis study^13^ was to evaluate repeatability of volume quantification in each of the sequences separately.

Given the definition of the ROIs, the volumes of each of the annotated regions for each combination of subject, time point and MRI sequence was measured by calculating the total number of pixels within a given region, and multiplying it by the volume of a single voxel. The latter was calculated by multiplying the in-plane pixel spacing and the distance between the consecutive image slices, as derived from the DICOM MRI data in the corresponding sequences. Mean ADC was automatically calculated as the arithmetical average voxel intensity over the segmented regions for the ADC images. These measurements were used to assess the repeatability of volume and mean ADC measurements in the study presented earlier^13^.

## Data records

### Overall approach to data encoding

The full dataset is archived with TCIA^21^ (Data Citation 1) and consists of the de-identified MR images and the data derived from the images. The latter group contains the ROIs, and the corresponding per-structure measurements derived from them. All of the components of the dataset are stored using the DICOM^17^ format. DICOM is the international standard adopted universally by the manufacturers of medical imaging equipment for storing their acquired images. In addition to modality-specific images, DICOM defines multiple types of image-related objects^20^, including the types of data in the present data descriptor.

DICOM objects are organized into a hierarchy of key-value items. The specifics of hierarchy, attributes to be included, constraints on their values, and other details are formalized by the DICOM Information Object Definitions (IODs), further referred to as “object types”. Those definitions can be applied to encode appropriate types of data into instances of DICOM object types that can be stored as DICOM files.

In the present dataset, we utilize three types of standard DICOM objects: the DICOM Magnetic Resonance Image (MR) produced by the imaging equipment, the DICOM Segmentation Image (SEG) for the labeling of voxels into the regions of interest (ROIs), and DICOM Structured Reports (SRs) that are encoded per the Measurement Report Template (Template ID, or TID) 1500 for the measurements derived from the segmented ROIs of the MR images.

DICOM objects that are encoded following IODs are called instances, and stored in files or sent over the network. One or more instances can be included in a single DICOM series, which in turn are organized into DICOM studies. Each of the DICOM instances, series and studies are assigned a unique identifier (UID) that are encoded as one of the dataset attributes. A DICOM study usually corresponds to a single imaging encounter of the patient. Individual series of a study correspond to different types of data acquired during that single encounter. In the present dataset, individual studies for a given subject (total of 15 subjects) correspond to the test-retest imaging studies done on different days (total of 30 studies). Series correspond to each of the different types of MR acquisitions of image data, and annotations of those acquisitions with the ROIs and measurements. Image series were generated by the imaging equipment, while the derived object series were created by the conversion tools provided in the *dcmqi* library^23^ (https://github.com/QIICR/dcmqi).

Each of the DICOM instances contains the instance-specific attributes, as well as the composite context attributes shared by all of the instances in a given series, study and for the same patient (in the remainder of this manuscript, we will refer to the DICOM attributes using the italicized *CameCase* notation). Such composite context attributes include, for example, *PatientID*, *PatientSex*, *PatientAge*, *PatientWeight*, *StudyDate* and *StudyTime*, and the *StudyInstanceUID* (UID of the study).

DICOM defines data models that link real-world objects (e.g., a patient, a study, a physical piece of equipment) with information objects defined by the standard (e.g., MR image or measurement report). Importantly, imaging-related evidence objects defined by the standard include such items as containers for image-derived measurements^24,25^ (including radiomic features^15,26^), and linking lab tests and pathology results with imaging evidence^27^. DICOM defines syntax rules for encoding of data and metadata, and includes controlled vocabularies that leverage widely used (extensible) external lexicons such as SNOMED CT^28^ (https://www.snomed.org/snomed-ct) and RadLex^29^. Both the well-defined syntax rules and controlled vocabularies are crucial to the successful extraction of knowledge from the data^30^. Every DICOM object has a unique identifier, and can include references to related (meta)data. DICOM Structured Reporting (SR)^31,32^ is the part of the standard intended to communicate imaging-derived findings, organized as hierarchical structures that link containers, codes describing concept names and values, references to related evidence, etc. DICOM SR “templates” define a pattern of content items and their relationships, constraining the general infrastructure and controlled terminologies for specific use cases. The DICOM Standards Committee has a process for introducing amendments via community participation. The DICOM standard is free to download and use without royalty or license fees. Based on these observations, we believe DICOM satisfies the Findable Accessible Interoperable Reusable (FAIR) guiding principles for scientific data management^33^.

### MR image data

The MR image data in this collection is encoded as instances of the DICOM Magnetic Resonance Image IOD^34^, as originally produced by the MR scanner, and then de-identified. DICOM MR images can be recognized by the value of the *SOPClassUID* attribute, which uniquely identifies them as stored instances of the DICOM Magnetic Resonance Image IOD, as well as the *Modality* attribute, which has a value “MR”. The traditional DICOM MR IOD encodes individual slices of a 3D volume as separate instances (files), which can be spatially-related for reconstruction into a volume using the *ImagePositionPatient* and *ImageOrientationPatient* attributes. The DICOM Enhanced MR Image IOD, which can encode all the slices of a volume in a single multi-frame object, was not used in this data set since that was not the form in which the data was acquired. Nor was it converted to the similar Legacy Converted Enhanced MR Image IOD^35^.

The MRI data was de-identified using a modified version of the Basic Attribute Confidentiality Profile defined by the DICOM in PS3.15 Appendix E.2^36^, following the standard operating procedures of TCIA^21,37^. In the process of de-identification, the original content of some of the attributes was completely removed (such as *PatientBirthDate*), while others were modified to preserve privacy of the subjects: *PatientName* and *PatientID* were modified to follow the “PCAMPMRI-nnnnn” pattern (for PCa mpMRI subject nnnnn), and the original dates were shifted by the same offset for all of the datasets.

The MR image data includes three types of images for each of the 30 studies. The type of image is indicated in the *SeriesDescription* attribute, which can have one of the following values corresponding to the acquisitions described above: “T2 Weighted Axial” (T2-weighted MRI acquisition with the slices oriented in axial plane of the patient), “Apparent Diffusion Coefficient” (image computed from the Diffusion-Weighted MRI trace image acquired with b-values of 0 and 1400 s/mm^2^, and “DCE Subtraction” (image computed from the DCE MR image as a pixel-wise difference between the early post-contrast and post-contrast phases of the acquisition, highlighting areas of contrast uptake). Technically, “Apparent Diffusion Coefficient” and “DCE Subtraction” images are derived, since they were obtained by post-processing of the imaging data obtained by the imaging equipment. However, they were derived by the software of the MRI scanner, and saved as MR images, per the normal imaging protocol followed at our institution.

The technical parameters of the MR acquisition protocol are stored in the MR-specific DICOM standard attributes, and include *MagneticFieldStrength*, *RepetitionTime*, *FlipAngle*, *EchoTime*, and *InversionTime*. The *Manufacturer* and *ManufacturerModelName* attributes can be used to distinguish the datasets obtained using the GE Signa HDx (subjects 1 through 7) and GE Discovery MR750w (subjects 8 through 15) scanners.

### Segmentations

Multiple ROIs were traced in each of the MR series, one for each structure, resulting in a labeling (segmentation) of image voxels belonging to the ROI. These segmentations were stored as DICOM Segmentation image objects^38^. Within the dataset, segmentations are recognized by their *SOPClassUID*, and have a value of “SEG” value for the *Modality* attribute. DICOM Segmentations store all slices of a segmentation within a single multi-frame object.

DICOM Segmentation image objects allow correspondence between the segmentations and individual MR images to be established in different ways. Informally, for the convenience of human users, we chose the *SeriesDescription* of the segmentation object to include the *SeriesDescription* of the segmented image, followed by “Segmentations”. Formally, the *ReferencedSeriesSequence* attribute of each segmentation object contains a list of instance UIDs (*ReferencedSOPInstanceUID*) of the source MR images. Also, correspondence between the individual frames of the segmentation and the individual source MR image slices (identified by their SOPInstanceUID) is encoded in the *SourceImageSequence* of each of the segmentation frames in *PerFrameFunctionalGroupsSequence*.

A single DICOM Segmentation instance may contain one or more segments. We encoded the segments of all of the ROIs corresponding to a single type of MR image (series) in a study as a single segmentation instance, because all of those structures were traced by the same reader during the same contouring session. Thus for each of the imaging studies, there is a single segmentation instance for each of the analyzed MR series. It contains multiple segments, one for each ROI.

Each segment encodes both the label assignment for the image voxels, and the structured metadata describing the content of the segment. Each segment’s metadata is described in an item of the *SegmentSequence* of the Segmentation IOD. To enable semantic interoperability of the data, DICOM uses codes from controlled terminologies to describe the anatomy and properties of segments. Such codes are defined as triplets consisting of the code value, the identifier of the coding scheme, and a human-readable meaning of the code. As an example, given the triplet (“T-D000A”, “SRT, “Anatomical Structure”), “T-D000A” is the code value, “SRT” indicates that this code belongs to the SNOMED CT terminology^28^, and “Anatomical Structure” is the human-readable code meaning. For historical reasons, DICOM currently uses the SNOMED ID rather than the Concept ID (of relevance, there are ongoing discussions within the DICOM community about the use of CT-style Concept ID in place of RT-style SNOMED IDs).

For each of the MR series, the following ROIs were segmented: the whole prostate gland, the whole peripheral zone of the prostate gland, and the suspected lesion (if and when a lesion was identified by the expert reader in a given study). In addition, ADC maps were annotated with the ROI of the normal peripheral zone tissue. The semantics of the segment are recorded as a combination of two coded attributes: *SegmentedPropertyCategoryCodeSequence* and *SegmentedPropertyTypeCodeSequence*. If the ROI defines a non-anatomical region, such as the extent of a lesion, the anatomic location of the segmented region is separately specified using the coded *AnatomicRegionSequence* attribute to define the location of the lesion, otherwise the anatomic location is used as the *SegmentedPropertyTypeCodeSequence*. The values we used for the individual segmented structures are summarized in Table 1.

Additional standard segmentation metadata recorded includes *SegmentAlgorithmType* attribute that in our case is always assigned value of “MANUAL” to indicate that a human performed the segmentation, and *RecommendedDisplayCIELabValue* with a CIELab color value for suggested display color for each segment.

The segments are stored in the *PixelData* attribute of the segmentation object as binary bit planes. We could have, but did not, reduce the size of each plane to a bounding box surrounding the object. We also could have, but did not, detect and exclude empty frames for the segment (corresponding to MR image planes outside of the ROI). The standard allows for either pattern. In our data, if the MR image series being annotated contains *n* instances (slices), each segment will also have *n* frames, each of which will have the same number of rows and columns as the image. Each of those frames is encoded using one bit per voxel, with values of one corresponding to the labeled voxels. Encoded frames for all of the segments are then concatenated to form the *PixelData* in a given Segmentation image instance. We did not use the Deflated Transfer Syntax to compress the resulting DICOM objects but simply encoded them in their uncompressed form. Encoding of the segmentations in the uncompressed form improves reusability of the resulting objects, since the specific compression scheme may not be supported by the recipient. Furthermore, the size of the segmentation objects in the uncompressed form is smaller than that of the segmented image series, limiting the practical need for compression.

We note that there are small numeric discrepancies (on the order of 0.001, in our case) in the *ImagePositionPatient* between the MR images and the corresponding segmentations. This discrepancy is due to fact that our DICOM conversion process utilized representation of the image segmentation stored using volumetric research format (NRRD) as input. That volumetric representation defines image geometry using origin, spacing, and directions, and assumes regular sampling of the volume. Representation of the source MR image being segmented encodes image origin for each of the image slices individually. From our experience, it is not uncommon to observe discrepancies on the order of 0.001 between the encoded *ImagePositionPatient* and the one calculated assuming regular sampling. We deliberately decided to accept this discrepancy, since the tool used for annotating the data utilized volumetric representation of the data (i.e., upon loading a scalar DICOM image series into 3D Slicer it is converted into volume, which defines the image matrix for the segmentation).

### Measurements

The ROI-based measurements are stored as DICOM Structured Reporting (SR) objects. DICOM SR organizes content hierarchically as a tree, each node of which is a content item. The constraints and required components of the hierarchy may be defined by a specific template. We followed the DICOM Measurement Report Template (TID 1500)^24^, in a manner similar to its previous application for encoding PET-derived measurements^20^. No non-standard attributes or content items were used.

A single SR object was created for each annotated MR image series. Within one SR object, individual measurements are grouped by ROI within the Imaging Measurements container, into Measurements Groups, as defined by DICOM SR TID 1411 Volumetric ROI Measurements, a subordinate template included by TID 1500. Individual measurements within a group are accompanied by the coded concepts defining the quantity measured, and the units of measurement, see Table 2. Volume measurement quantity is defined by the code (“G-D705”, “SRT”, “Volume”), with coded units (“mm3”, “UCUM”, “cubic millimeter”). “UCUM” is the DICOM coding scheme designator for the externally defined standard Unified Code for Units of Measure^39^. Mean ADC quantity is (“113041”, “DCM”, “Apparent Diffusion Coefficient”), post-coordinated with the derivation (“R-00317”, “SRT”, “Mean”), and units of (“um2/s”, “UCUM”, “um2/s”).

**Figure 1:**
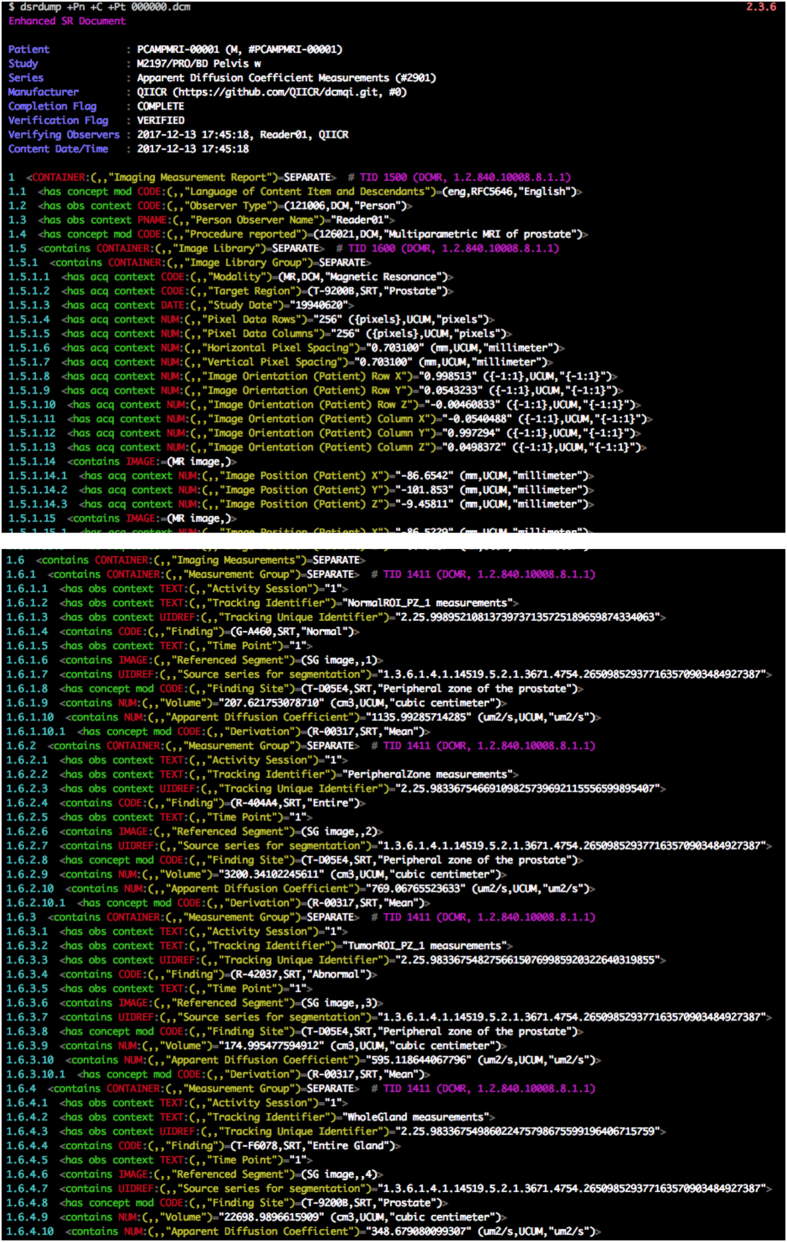
An abbreviated excerpt of the human-readable view of the content tree for one of the measurements SR object. The example corresponds to the ADC series for subject PCAMPMRI-00001, baseline study, illustrating encoding of the measurement report header (top portion of the figure) and measurements for one of the measurements group (bottom portion of the figure). In the top sub-figure, “Image Library Group” container is abbreviated to exclude the repeated items corresponding to the individual MR image instances. The text is annotated in color to identify specific components of the report: content item type is in red, relationship of a content item type to the parent is in green, code triplet of the content item concept in yellow (note that Code Value and Coding Scheme Designator are omitted for brevity, only Code Meaning is shown), value of the content item in white. Text in purple references the specific templates of the standard instantiated within the document: this instance uses templates TID 1600 “Image Library” and TID 1411 “Volumetric ROI Measurements” subordinate to TID 1500. The view was produced using the DCMTK dsrdump tool.

In addition to storing the individual measurements, each measurement group contains metadata described by the following coded concepts, as defined by the template (also see Fig. 1 for the illustration of how those concepts are populated on the example of one of the SRs included in the dataset):

- *“Time Point”*: this content item is set to either 1 or 2, to differentiate between the baseline and repeat imaging exam;
- *“Tracking Unique Identifier”*: a DICOM UID^40^ of the measurement group. This UID defines linkage of measurements associated with the same structure in different contexts, temporal or otherwise. Thus a particular ROI identified at one time point and on one image series may be associated with an ROI corresponding to the same structure on a different image series for the same time point, or at a different time point. In our dataset, Tracking Unique Identifier is identical for a given structure across acquired series in the studies within and across time points for the measurement groups associated with the whole prostate gland, whole peripheral zone and individual lesions. Tracking Unique Identifiers are different in the baseline and repeat studies for the normal region ROIs, however, since the reader did not attempt to localize the same normal tissue region, only to choose a representative region.
- *“Tracking identifier”*: a free text human-readable analog to the Tracking Unique Identifier, which although not required to be this way in the standard, serves in this dataset as the unique name of the measurement group and can be used to easily identify the structure on which the measurements were performed.
- *“Finding Site”* and *“Finding”*: contain SNOMED CT codes describing the structure being measured. The Finding Site defines the anatomical location, and the Finding defines whether or not the entire structure (prostate or peripheral zone) or part of the structure (only normal peripheral zone) or a lesion was measured. The values we used are summarized in Table 3. These correspond to the coded descriptions of the explicitly referenced segments in the Segmentation instance, though the specific codes and their pattern of use reflect historical differences in the evolution, design and intent of the SEG and SR objects and templates, as well as the particular distinctions that are relevant for our purposes.
- *“Source series for segmentation”*: the SeriesInstanceUID of the DICOM MR image series that was segmented.
- *“Referenced Segment”*: the *SOPInstanceUID* of the DICOM segmentation object and the segment number defining the ROI used to perform the measurement.

In addition to measurements derived from images, the SR document contains several other components specified in the template definition. Image Library container (defined by DICOM SR TID 1600) contains key content items describing the image from which the measurements were derived, and allows the SR to be understood in context without the need to dereference other objects in the dataset. Image Library content items include the imaging modality, geometric information for the individual image slices, and a coded information about the target region (“T-9200B”, “SRT”, “Prostate”).

As with other DICOM objects, the composite context of the DICOM SR objects is encoded at the attribute level (outside the SR content tree).

## Technical validation

**Figure 2:**
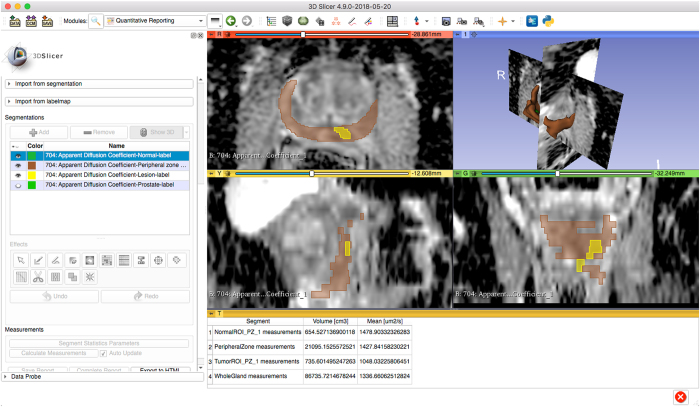
An example visualization of a single annotated image using 3D Slicer software. Shown is the ADC image for subject PCAMPMRI-00008, baseline study. The visualization corresponds to the same image as shown in Fig.2c of the analysis manuscript^13^. The visualization was tested using the nightly release of 3D Slicer downloaded on May 20, 2018.

The dataset presented here was previously analyzed to evaluate repeatability of quantitative MR parameters (ROI volume and mean ADC value)^13^. Technical validation of the correctness of the conversion into the DICOM representation was confirmed as follows. First, we randomly selected a subset of encoded segmentations, and visually confirmed the consistency of the segmented ROIs as loaded from the DICOM representation and the non-DICOM format used for the data capture originally. For this purpose we used the 3D Slicer QuantitativeReporting module^22,23^ (see Fig. 2 for an example visualization), which allows loading and display of image segmentations from DICOM SEG objects overlayed on the annotated image, and displays the associated segmentation-based measurements loaded from the corresponding DICOM SR objects. We also confirmed the agreement between the ROIs volume and mean ADC values as loaded from DICOM SR and the values used in the conversion. Second, we have confirmed that the implementation we used for conversion of the segmented ROIs into DICOM representation is consistent with the independent implementations, as part of the DICOM4QI demonstration and connectathon^41^ (https://dicom4qi.readthedocs.io). Finally, we validated the compliance of the encoding of the objects with the DICOM standard by using the *dciodvfy* tool included in the *dicom3tools* software (http://www.dclunie.com/dicom3tools.html). SR objects were further validated using the *com.pixelmed.validate.DicomSRValidator* tool of the *Pixelmed* toolkit (http://pixelmed.com/#PixelMedJavaDICOMToolkit) to verify their compliance with the TID 1500 template structure.

## Usage notes

The dataset is available in the limited-access QIN-PROSTATE-Repeatability collection of The Cancer Imaging Archive (TCIA) (Data Citation 1). Limited access was requested by the Institutional Review Board (IRB) of the Brigham and Women’s Hospital, requiring that interested parties apply for access and agree to the terms of use before accessing the data. The main reason for restricted access was in order to maintain the list of individuals accessing the dataset per the requirements of the BWH IRB.

The dataset can be explored using a number of publicly available tools. Individual image series, segmented regions and the measurements corresponding to those regions can be visualized using 3D Slicer software^22^ (http://slicer.org) with the QuantitativeReporting extension installed^23^. Fig. 2 shows an example of such visualization rendering of the annotated regions and measurements for the ADC series in one of the subjects. DICOM SEG visualization is also supported by a growing number of publicly available and commercial tools, including MITK^42^, MeVisLab (https://www.mevislab.de/), ePAD^43^, and several others discussed on our public DICOM4QI iteroperability connectathon web pages^41^ (https://dicom4qi.readthedocs.io). Conversion of the DICOM SEG format into any of the commonly used volumetric research formats supported by the Insight Toolkit^44^, such as NIfTI (http://nifti.nimh.nih.gov/) or NRRD (http://teem.sourceforge.net/nrrd/) can be done using converters from the freely available *dcmqi* software^23^. Individual metadata attributes can be extracted into tab-delimited tables linked by SOPInstanceUID using the freely available software (https://github.com/QIICR/dcm2tables). Individual DICOM files can be explored at the attribute level using *dcmdump* tool included in the open source DCMTK software^45^. DICOM SR documents can be examined at the level of the SR content tree using *dsrdump* tool of DCMTK. Both *dcmdump* and *dsrdump* tools are wrapped in the interactive *dicom-dump* package of the freely available Atom editor (https://atom.io/packages/dicom-dump).

We note that contrary to the standardized approach we have taken here, it is common in the imaging research community to use *ad hoc*, proprietary or other non-standard formats for communicating data derived from images. However, such approaches inevitably rely on domain- or site-specific conventions, formats and software (if available), which makes harmonization of different types of data and datasets obtained from different sources difficult if not impossible. Furthermore, such approaches do not meet the Findable, Accessible, Interoperable, and Reusable (FAIR)^33^ principles of data management. Examples of the non-FAIR aspects of research formats include: non-standard approaches to sharing data typically do not utilize unique identifiers for the derived datasets; they have limited capabilities and conventions for associating and encoding metadata with the data it describes; and they do not reuse existing structured terminologies to describe the metadata or its values, limiting semantic interoperability of the data. The need of wider utilization of the existing standards for data harmonization in medical research, including algorithm comparison challenges^46^, is widely recognized^47,48^. While understanding and adopting the DICOM format can be challenging in practice, it establishes a common framework for data management in the imaging domain. Although the use of DICOM for communicating the derived data objects shared in this data descriptor may seem excessive and overly burdensome, we argue that it maximizes interoperability of the data for machine readability, harmonization with other datasets, and the (unforeseen) future uses. There are no viable existing alternative approaches to encoding the types of data we share that are known to us that satisfy the FAIR guidelines, though there are some academic efforts that lay claim to the word “standard”. We stress that the supporting tools we have described, and others, are publicly available, which enable conversion of the shared DICOM datasets into alternative research representations to accommodate the needs of data consumers without native support of the relevant DICOM object types in their workflows. Of course, converting DICOM to research formats will often be lossy with respect to metadata if the research format provides no support to represent it.

We believe the effort of creating and consuming standardized datasets, such as the one developed in the present data descriptor is considerably lower than it used to be with the availability of the open source tools discussed in the paper. The foremost requirement for creating a standardized DICOM representation of the analysis results is the availability of the source DICOM images being analyzed. Given such representation is available, the conversion process should be straightforward and can be done in batch mode using *dcmqi*. One of the challenges in implementing the conversion process is the selection of the standard codes to describe the metadata concepts required by the standard representation. Ideally, those should be selected from existing ontologies to enable semantic interoperability of the data. The complexity of identifying such codes varies based on the specific use case. As an example, identifying codes describing human anatomy could be accomplished with the SNOMED-CT or FMA ontologies. However, identifying ontologies and terms for describing concepts that are exploratory in nature (e.g., radiomics analyses, covariates of the perfusion analyses) can indeed be challenging. We note however, that DICOM permits the use of “private” coding schemes populated by the entity creating the document, which is straightforward, but limits the semantic interoperability of the data. The details on the selection of the terms is provided in the *dcmqi* documentation. The exact amount of effort required will obviously vary from dataset to dataset, but we would argue that the process can be expected to become easier over time as more datasets are published in the standard form. In addition we note that the process of identifying the proper metadata coding should be considered a necessary aspect of data publication to achieve FAIR representation, and should therefore be not a burden but a requirement.

The manuscript is a data descriptor that accompanies our earlier publication in *Investigative Radiology*^13^ and does not describe the analysis framework used therein. The prior publication utilizes a commonly used framework for evaluating repeatability of measurement using Repeatability Coefficient and Intraclass Coefficient. Similar approaches have been used in other studies investigating repeatability of radiomics features (e.g., see the study by Hu *et al.*^49^), and can be applied to the presented dataset. We have also published initial analysis of radiomics features based on the presented dataset as a preprint, see Schwier *et al.*^50^ The framework for the evaluation of repeatability is thus well-established. We are not claiming that the dataset will allow comprehensive evaluation of the performance of an imaging biomarker. High repeatability does not imply good performance evaluating the disease or clinical utility. Rather, repeatability evaluation can serve as a component in the overall evaluation of a biomarker. The presented dataset alone cannot be used to make statistically-sound claims about performance of analysis approaches. Our intent is to provide a dataset that could supplement a larger dataset and support evaluation of an analysis method in a test-retest setting. While the dataset is small, this is due to the inherent difficulty in conducting test-retest studies (e.g., the study that collected this dataset approached over 180 subjects over the course of two years, with only 15 subjects completing the protocol). Other studies that investigated mpMRI repeatability in the prostate reported similar-sized cohorts: 8 in Gibbs *et al*.^51^, 10 in Litjens *et al.*^9^, 14 in Sadinski *et al.*^11^ Notably, neither of the aforementioned studies is accompanied by a publicly available dataset. In absence of evaluations that utilized larger cohorts, those small sample studies can still be valuable. As an example, claims stated in the Quantitative Imaging Biomarker Alliance (QIBA) Diffusion Weighted Imaging profile^52^ are based on the analysis of those small cohorts. As another example, the seminal study by Aerts *et al.*^53^ utilized the lung CT RIDER test-retest dataset consisting of 31 subjects as part of a supervised feature selection methodology. Examining proceedings of MICCAI 2018, the premier conference concerned with innovation in medical imaging computing, there are a number of studies that utilize cohorts of less than 20 subjects for evaluating models trained on larger populations. These are just some of the examples illustrating the value of datasets that are relatively small in size.

We envision a number of applications where the presented PCa dataset could be valuable. Characterization of disease using radiomic phenotyping^15^, including in prostate cancer^3,4^, has recently been explored by a number of investigators. Stability of radiomic features is often used for unsupervised pre-selection of radiomic features in developing signatures of the disease^53^. Although there are publicly available test-retest datasets for such diseases as lung cancer^14^, we lack data on repeatability of radiomic features in prostate mpMRI (see our initial work in this direction by Schwier *et al*.^50^). Computer-aided approaches for automated detection and characterization of PCa have been proposed^8,54^, but again, consistency of such automated processing results in a test-retest setting has not been evaluated. Furthermore, the dataset can be utilized to enable investigation and testing of new image computing tools that can support tasks related to image quantitation. As an example, it could be used to evaluate consistency of automated segmentation of the structures of interest (e.g., numerous approaches to automated prostate segmentation have been proposed^55,56^), to evaluate the effect of automated image registration techniques on improving stability of the extracted quantitative measures, or to investigate how different approaches to normalization of the MR signal^57,58^ can affect the consistency of the radiomic features. Importantly, due to the nature of the study, although annotations were performed by an expert, we do not know the exact boundaries of the manually traced structures. As such, the dataset cannot be used to evaluate accuracy of PCa detection or precision of boundary localization, though it can be used to investigate consistency of segmentation across multiple readers in a secondary analysis. We also envision that it might be valuable to perform a study annotating the dataset with the both baseline and repeat images being available to the reader, which we expect should improve the consistency of localization of the boundaries of the annotated regions, potentially leading to the improved repeatability of the measurements.

We note the dataset does not include the PI-RADS v2 interpretations of the findings, since the standardized machine-readable definition of PI-RADS v2 reports in DICOM is currently not available. We plan to augment the dataset with the structured reports encoding PI-RADS findings when such definition becomes available, but it is out of scope for the present data descriptor.

## Additional information

**How to cite this article**: Fedorov, A. *et al*. An annotated test-retest collection of prostate multiparametric MRI. *Sci. Data*. 5:180281 doi: 10.1038/sdata.2018.281 (2018).

**Publisher’s note**: Springer Nature remains neutral with regard to jurisdictional claims in published maps and institutional affiliations.

## Supplementary Material



## Figures and Tables

**Table 1:** Per-segment coded attributes defining semantics of the segmented ROI.

Segmented structure	SegmentedPropertyCategoryCodeSequence	SegmentedPropertyTypeCodeSequence	AnatomicRegionSequence
*Prostate gland*	(“T-D000A”, “SRT, “Anatomical Structure”)	(“T-9200B”, “SRT”, “Prostate”)	NA
*Peripheral zone of the prostate gland*	(“T-D000A”, “SRT, “Anatomical Structure”)	(“T-D05E4”, “SRT”, “Peripheral zone of the prostate”)	NA
*Lesion in the peripheral zone of the prostate gland*	(“M-01000”, “SRT”, “Morphologically Altered Structure”)	(“M-01100”, “SRT”, “Lesion”)	(“T-D05E4”, “SRT”, “Peripheral zone of the prostate”)
*Normal tissue in the peripheral zone of the prostate gland*	(“T-D0050”, “SRT, “Tissue”)	(“G-A460”, “SRT”, “Normal”)	(“T-D05E4”, “SRT”, “Peripheral zone of the prostate”)

**Table 2:** Coded terms used for encoding of the measurements derived for the annotated regions of interest.

Measurement	Quantity code	Quantity modifier concept	Quantity modifier value	Units code
*Volume of the region of interest*	(“G-D705”, “SRT”, “Volume”)	N/A	N/A	(“mm3”, “UCUM”, “cubic millimeter”)
*Mean Apparent Diffusion Coefficient (ADC) for the region of interest*	(“113041”, “DCM”, “Apparent Diffusion Coefficient”)	(“121401”, “DCM”, “Derivation”)	(“R-00317”, “SRT”, “Mean”)	(“um2/s”, “UCUM”, “um2/s”)

**Table 3:** Structured report content items defining semantics of the measurements on the referenced ROI.

Measured structure	Finding	Finding Site
Prostate gland *	(T-F6078, SRT, “Entire Gland”)	(“T-9200B”, “SRT”, “Prostate”)
*Peripheral zone of the prostate gland*	(R-404A4, SRT, “Entire”)	(“T-D05E4”, “SRT”, “Peripheral zone of the prostate”)
*Lesion in the peripheral zone of the prostate gland*	(R-42037, SRT, “Abnormal”)	(“T-D05E4”, “SRT”, “Peripheral zone of the prostate”)
*Normal tissue of peripheral zone of the prostate gland*	(“G-A460”, “SRT”, “Normal”)	(“T-D05E4”, “SRT”, “Peripheral zone of the prostate”)
